# A Depression Prediction Algorithm Based on Spatiotemporal Feature of EEG Signal

**DOI:** 10.3390/brainsci12050630

**Published:** 2022-05-11

**Authors:** Wei Liu, Kebin Jia, Zhuozheng Wang, Zhuo Ma

**Affiliations:** 1 Faculty of Information Technology, Beijing University of Technology, Beijing 100124, China; liuwei0823@bjut.edu.cn (W.L.); wangzhuozheng@bjut.edu.cn (Z.W.); mazhuo@emails.bjut.edu.cn (Z.M.); 2 Beijing Laboratory of Advanced Information Networks, Beijing 100124, China; 3 Beijing Key Laboratory of Computational Intelligence and Intelligent System, Beijing University of Technology, Beijing 100124, China

**Keywords:** depression prediction, spatiotemporal features, deep learning, EEG signals, neural network

## Abstract

Depression has gradually become the most common mental disorder in the world. The accuracy of its diagnosis may be affected by many factors, while the primary diagnosis seems to be difficult to define. Finding a way to identify depression by satisfying both objective and effective conditions is an urgent issue. In this paper, a strategy for predicting depression based on spatiotemporal features is proposed, and is expected to be used in the auxiliary diagnosis of depression. Firstly, electroencephalogram (EEG) signals were denoised through the filter to obtain the power spectra of the three corresponding frequency ranges, Theta, Alpha and Beta. Using orthogonal projection, the spatial positions of the electrodes were mapped to the brainpower spectrum, thereby obtaining three brain maps with spatial information. Then, the three brain maps were superimposed on a new brain map with frequency domain and spatial characteristics. A Convolutional Neural Network (CNN) and Gated Recurrent Unit (GRU) were applied to extract the sequential feature. The proposed strategy was validated with a public EEG dataset, achieving an accuracy of 89.63% and an accuracy of 88.56% with the private dataset. The network had less complexity with only six layers. The results show that our strategy is credible, less complex and useful in predicting depression using EEG signals.

## 1. Introduction

According to the World Health Organization (WHO), depression is one of the major disabilities causing mental disorders that affects around 322 million people, accounting for 4.4% of the world’s population [1,2,3]. Depression not only leads to a series of physical problem but also has a potentially high risk of suicide, which aggravates the burden on patients, their families and society [4]. The diagnosis of depression is mainly based on the 10th revision of the International Classification of Diseases (ICD-10) developed by WHO [5] and the 5th revision of the Diagnostic and Statistical Manual of Mental Disorders (DSM-5) established by the American Psychiatric Association (APA) [6]. At present, the diagnosis of depression is still mainly based on the chief complaint of clinical symptoms, and there is no specific laboratory or auxiliary diagnostic method. Due to a lack of awareness, unskilled health-care practitioners, lack of resources, and inaccurate diagnoses, a shocking 50% of persons with depression remain untreated. This disorder can be treated easily if diagnosed in a timely manner and when diagnosed properly, so there is an urgent need to develop a clear understanding of the etiology and pathogenesis of the disease, and to find an objective and effective method with which to identify depression.

EEG, electrocardiography (ECG) and magnetic resonance imaging (MRI) have been used for the diagnosis of various diseases. EEG has been explored as an effective biomarker and diagnosis tool for the detection of neurological disorders such as depression, epilepsy, seizure, Alzheimer’s, Parkinson and in the analysis of emotions in comparison to others because of its non-invasive and economical nature [7,8,9,10,11,12,13,14,15,16]. Research shows that different frequency ranges and the spatial distributions of EEG are associated with different functional states in the brain [17]. Various brain rhythms have been generalized, along with their frequency ranges, regions of occurrence and characteristics [18,19,20,21]. An analysis of EEG signals with different frequencies will help physicians to diagnose and identify patients with depression effectively. Other research studies have used some brain rhythms depending upon the requirement of certain frequency bands for the analysis of the particular application. For example, Beta can be used to classify the sleep stage, Alpha is used in the study of emotion recognition, and both Alpha and Beta have been used in dementia studies [22]. Alpha, Beta and Theta have been used for depression or stress recognition [23] as the amplitude and frequency rhythm are related to differences in the brain function between patients and healthy people. Koller-Schlaud et al. [24] discovered that in the resting state, theta activity at the central electrode site is highly distinct and were able to discriminate between healthy controls and bipolar depressed controls using an analysis of variance model. Kang et al. [25] found that the greatest performance for the classification model for diagnosing depression was achieved using alpha asymmetry images. For depression patients, Liu et al. [26] found a substantial link between the long-distance edge of the Beta band, which was distributed largely within the frontal brain areas and between the frontal and parietal-occipital brain areas. Grin-Yatsenko et al. (2010) [27] also found increased activity in theta, alpha and beta bands in occipital and parietal areas of the brain of depressed subjects.

According to the frequency property of EEG signals, we preprocessed the original signals and then used the deep learning framework to realize the automatic prediction of depression which in turn could help more people to identify depression as early as possible.

EEG is the overall response of the electrophysiological activity of human brain nerve cells in the cerebral cortex or scalp surface, which comprehensively reflects the functional state of the brain and contains a high amount of physiological and disease-related information. Therefore, it is important for understanding how the human brain processes information and in the diagnoses of mental diseases, and we can complete the diagnosis and treatment of neurological diseases by detecting and recording human EEG signals [28]. Compared with CT and MRI, EEG has a higher temporal resolution [29]. EEG is a valuable research and diagnostic tool, especially when specific studies require millisecond-level temporal resolutions, such as those on anxiety, psychosis and depression [30]. Since EEG data are graphically represented, researchers often use AI-based models to analyze such data [31,32,33,34]. For example, Field and Diego [35] used a linear discriminant analysis to process EEG data and achieved 67% accuracy in classifying normal and depressed patients. In addition, Iosifescu et al. [36] used a support vector machine (SVM) to classify resting-state EEG data from the 8-lead midpoint on the forehead of 88 subjects, and achieved a classification accuracy of 70%. Bisch et al. [37] used logistic regression (LR) to classify 9-lead EEG data of depression, with an 83.3%classification accuracy. Although EEG can be used to simplify the data-collection process, it leads to information loss. More importantly, the presence of a large number of untapped factors in EEG data can lead to a large amount of noise in classification decisions. Therefore, the development of machine learning models that are more suitable for EEG data will become the main research direction in the future.

Numerous studies have shown that there are significant differences in brain activity between people with depression and healthy people. For example, the EEG signals of patients with depression are significantly different in amplitude, energy and other indicators compared with healthy people [38]. Ahmadlou et al. presented a model with the combination of the Wavelet-Chaos method, Higuchi’s–Katz’s Fractal Dimension (HFD–KFD) and Enhanced Probabilistic Neural Network (EPNN) for the diagnosis of Major Depression Disorder (MDD) with an accuracy of 91.3% [39]. Hosseinifard et al., extracted non-linear features and used the Logistic Regression Classifier to differentiate normal and depressed classes and attained an accuracy of 90.05% [32]. Faust et al., performed wavelet packet decomposition on EEG signals and extracted non-linear features and entropy features. Then, these features were fed into a Probabilistic Neural Network (PNN) classifier to categorize normal and depressed patients, which reported an accuracy of 98.20% [40]. Bairy et al. used a combination of wavelet entropies, energy features and a Support Vector Machine classifier with a Radial Basis Kernel Function (SVM RBF) that reported an accuracy of 88.9% [41]. All the mentioned studies used handcrafted features, and the selection of an appropriate feature set is a very complex task. Acharya et al. proposed a deep learning-based 13-layer CNN model [42] and Ay et al., developed an 11-layer CNN-LSTM model to automatically classify normal and depressed patients with much better accuracy [43]. Additionally, Geetanjali Sharma et al., proposed a Depression Hybrid Neural Network that is accurate, less complex and uses CNN, windowing and LSTM [44].

To summarize, the initial research mainly relied on handcrafted feature extraction such as wavelet entropies, DWT, etc. [34,39,40,41], but the process of manual extraction affects the final classification results of the model. The CNN model proposed by Acharya has the advantage of automatic feature extraction [42] and has a higher accuracy than previous models. It uses 13 neural layers. The addition of the LSTM network to the CNN network by Ay et al., achieved better results [43,44].

In this paper, a method for depression prediction based on spatial and temporal characteristics is proposed.

1. In signal preprocessing, the signal frequency domain information, the space domain information between the electrodes of the acquisition equipment, and timing characteristics are fully utilized. The extraction of features with this strategy can be implemented automatically without manual acquisition. The model explores the GRU network with CNN layers whereby the CNN layers extract features and the GRU block provides sequence learning.
2. A model was proposed with relatively few layers (6 layers), and consequently, a relatively low level of complexity.

The rest of this article is organized as follows. In Section 2, the dataset and the proposed framework are explained. In Section 3, the results of this study are reported. Section 4 includes a discussion. Additionally, the conclusion is provided in Section 5.

## 2. Materials and Methods

### 2.1. Subjects

A public dataset provided by H. Cai et al., (MODMA dataset [45]) was utilized to evaluate the proposed method of depression prediction. The dataset was published by the UAIS laboratory of Lanzhou University in 2020, which contains EEG data from patients with clinical depression, as well as data from normal controls. Before the experiment, the self-reported Patient Health Questionnaire-9item (PHQ-9) and Generalized Anxiety Disorder-7 (GAD-7) were self-rated by all subjects. All patients were carefully selected by the hospital’s professional psychiatrist. The EEG dataset includes 128 channels of resting EEG signals collected from 53 subjects using HydroCel Geodesic Sensor Net (HCGSN). The 53 participants included 24 major depressive patients and 29 normal controls. The sampling rate was 250 Hz.

A further private data set was provided by the psychiatric department of a 3Agrade hospital in China, and was used to verify the effectiveness of the model, which enrolled 32 subjects,16 of which had a medically confirmed diagnosis of depression. The EEG signals were recorded with 16 cup electrodes mounted on a special cap in the following positions: Fp1-Fp2, F3-F4, F7-F8, C3-C4, T3-T4, T5-T6, P3-P4, O1-O2 according to the international 10–20 system. The sampling rate was 100 Hz. All participants were asked to remain in the resting state in a quiet room with their eyes closed and awake, a process which required 4 min. Labels for classification were assigned according to the presence or absence of depression diagnosis. In cases of missing data, the label was derived from the BDI result according to whether it exceeds the minimal range (score of 1–13). Table 1 describes the two datasets in detail.

### 2.2. Proposed Classification Method

**Figure 1:**
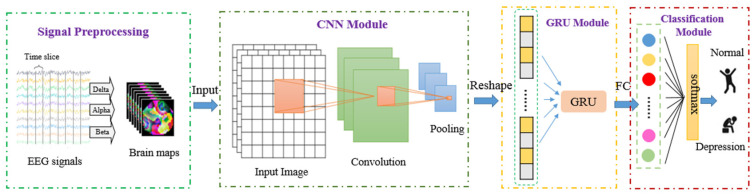
Proposed approach for depression prediction.

**Figure 2:**
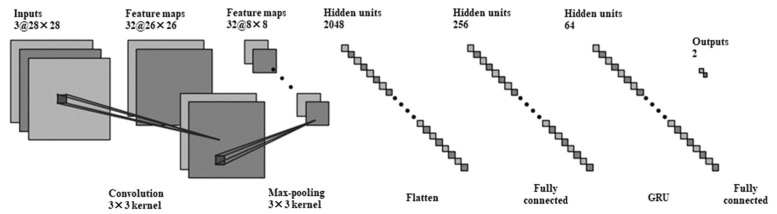
The network structure and parameters of the strategy.

Many previous studies demonstrate that deep learning and EEG can be used to recognize depression [46,47]. In order to prevent mild depression from worsening into moderate or major depression, we should pay more attention to mild depression and detect early symptoms of depression. As we know, EEG signals communicate spatial information, but this information has rarely been considered. Figure 1 illustrates the overview of the proposed framework for predicting depression. As shown in Figure 1, the proposed method contains EEG signal preprocessing, CNN, GRU and classification steps. In the first step, the spatial and frequency information of EEG signals was extracted to generate brain map sequences. Next, the CNN module was used to extract the feature automatically, and then the GRU module was connected to learn sequential information. The network structure and parameters of the strategy in this paper are shown in Figure 2. Finally, the classification module was trained and validated with the features that were extracted in the previous steps and contained spatial, frequency and temporal information.

#### 2.2.1. EEG Signal Preprocessing

Since there were only 53 or 32 raw data samples, the classification was not ideal in this case. Therefore, the intercepted data were divided into 10 segments of 1 s. Finally, we obtained 530 and 320 samples.

The process of EEG signal acquisition may be interfered with by careless human operation, an external environment interference, and electromagnetic interference of the device itself, which may lead to different types of noise in the collected data. While the amplifier in acquisition equipment can reduce the influence of some interference noises, there are still many artifacts such as eye blinks and movement, muscular activities, channel noise and power line noise. Therefore, EEG signals need to reduce the noise and suppress destructive artifacts with preprocessing. During the recording, a 0.5 Hz high-pass filter, a 100 Hz low-pass filter and a 50 Hz notch filter were considered to remove the low-frequency noise, irrelevant signals and the baseline noise from the data, respectively. The fast independent component analysis (Fast ICA) algorithm was used to calculate independent components of the filtered signals to remove artifacts resulting from muscles and eye movements within the EEG signals. The main steps of Fast ICA were as follows:

- The EEG signal was processed by Fast ICA to obtain several independent components whereby the independent components include the independent component containing the EEG artifact and the independent component without the EEG artifact;
- Wavelet transforms and the differential evolution algorithm were used to process the independent component containing the artifact to obtain the artifact component;
- Based on wavelet reconstruction and inverse transformation, an EEG signal was obtained to remove the artifacts according to the artifact component.

After removing the artifacts, the signal was cut at one second to extract the feature of EEG signals.

**Figure 3:**
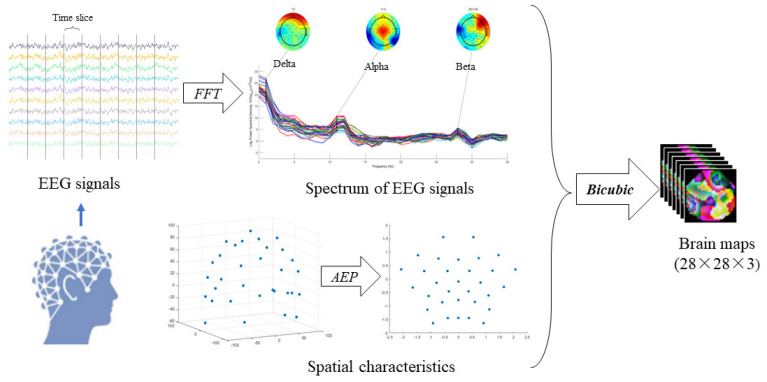
The preprocessing of EEG signals.

The potential activity of electrodes at different spatial sites has a correlation rule in the analysis of EEG signals, indicating synchronous and asynchronous electrical activity of the cerebral cortex potential. As a result, spatial characteristics are extremely useful in the character analysis of EEG signals. EEG is a sequence of time series collected on the scalp at different spatial locations, so the spatial characteristics of EEG signals can be obtained by mapping the position of its electrodes from a three-dimensional space onto a two-dimensional surface. The spatial characteristics are acquired using Azimuth Equidistant Projection (AEP) [48]. Research [22] shows that there are considerable changes in the θ (4–8 Hz), α (8–13 Hz) and β (13–30 Hz) spectrums between patients with depression and healthy people. Therefore, by extracting the θ, α and β spectrum of EEG signals, respectively, and using Bicubic interpolation we were able to obtain three brain maps [49], which contain frequency domain information. Then, the three images were superimposed to produce a new brain map as the last step of signal preprocessing. The new brain map sequences will be the input to the next model, containing temporal, frequency and spatial information of EEG signals. Figure 3 depicts a schematic diagram of EEG signals preprocessing.

#### 2.2.2. Extraction Using CNN

**Figure 4:**
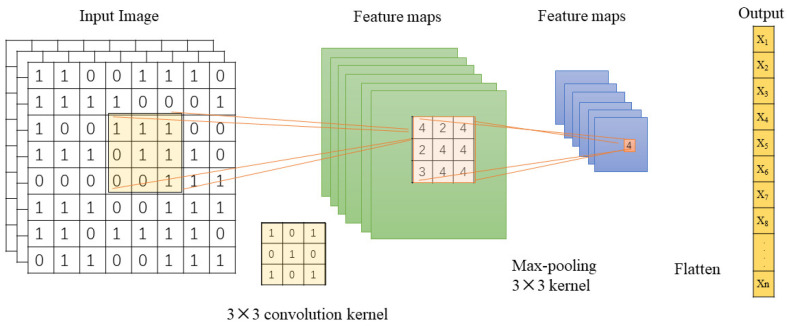
The Convolution Process.

CNN is a type of feed-forward neural network with convolutional computations and a deep structure. The convolution process is performed by sliding the specific kernels over the input data to obtain the feature map. The convolved output is generated using the following equation:

(1) g(x,y)=f(x,y)∗C(u,v)

where *g*, *f* and *C* denote the output feature map, input data and filter, respectively. The process is depicted in Figure 4.

The CNN has representational learning ability and can classify the input information according to its hierarchical structure. In particular, the brain map sequences, which were the results extracted with EEG signal processing as input signals have abundant spatial and frequency domain information. Additionally, CNN can amplify the difference in input signals. In our work, the input of the convolution layer was 28 × 28 × 3 and the convolution kernels’ size was 3 × 3 × 3 with 32 filters. We employed the Leaky Rectified Linear Unit (Leaky ReLU) as the activation function because it can speed up the learning ability and improve classification accuracy. A max-pooling layer with a kernel size of 3 × 3 was applied to reduce data sensitivity and computational complexity on the basis of reserving the data information. As shown in Figure 2 (CNN module), after the convolution calculation, we obtained a sequence of one-dimensional vectors(x_1_~x_n_) containing temporal, frequency and spatial information for space–time features.

#### 2.2.3. Learning Model with GRU

**Figure 5:**
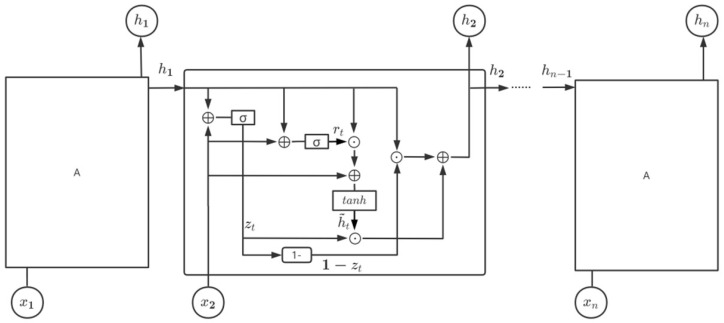
Structure of GRU.

The CNN model can automatically extract features, but it cannot feedback the output to the network and the model has poor performance when learning time-series information. EEG-signal classification is a typical sequence model task. As shown in Figure 2 (GRU module), we proposed a hybrid model that uses CNN and GRU at the same time. The GRU architecture was proposed by Cho et al. [50] in 2014 and is simpler and requires less time than LSTM. The specific implementation of this model is shown in Figure 5, which has specific hidden units called memory cells that are used to remember the previous input for a long period of time.

Figure 5 shows that, at the t time step, there are two kinds of gate operations in one hidden node of GRU, namely the update gate *z_t_* and the reset gate *r_t_*. Similar to LSTM, the currently hidden output *h_t_* is computed based on the current input *x_t_* and the previously hidden output *h_t_*_−1_.

(2) $$r_{t}=\sigma \left(W_{r}x_{t}+U_{r}h_{t-1}+b_{r}\right)$$

The update gate is expressed as follows:

(3) $$z_{t}=\sigma \left(W_{z}x_{t}+U_{z}h_{t-1}+b_{z}\right)$$

The hidden state (memory) is presented as follows:

(4) $$\{\begin{array}{c} h_{t}=\left(1-z_{t}\right)⨀h_{t-1}+z_{t}⨀{\tilde{h}}_{t}\\ {\tilde{h}}_{t}=\tanh\left[Wx_{t}+U\left(r_{t}⨀h_{t-1}\right)+b\right] \end{array}$$

In Equations (2)–(4), *W_r_*, *W_z_*, *W* and *W*_0_ are the weight matrices of GRU neural networks related to the input *x_t_*; *U_r_*, *U_z_* and *U* are the weight matrices of GRU neural networks related to hidden state hte1; *b_r_*, *b_z_*, *b* and *b*_0_ are the biases; *x_t_* is the input vector; the operation ⨀ stands for the Hadamard product; σ represents the logistic sigmoid function, and tanh represents the hyperbolic function.

Then, the output from the fully connected layers to the output layer uses Leaky ReLU as the exponential linear unit. To avoid overfitting, the dropout layer is introduced. The output layer chooses softmax as a classifier to classify the output.

#### 2.2.4. Validation

To validate the reliability and generalization of classifiers and datasets, an independent test was used in this paper. For the independent dataset test, each dataset was divided into two parts, a training set and a testing set. Two-thirds of the samples were chosen randomly and assigned as the training set and the remainder were used in the testing set. The Leave-One-Out Cross-Validation (LOOCV) method was applied in the classification of training data and the genetic algorithm was applied for feature selection. The results of classifiers on the test datasets are shown in Section 3.3.

The experimental environment was an Inter(R) Core (TM) i7 processor, with 16G memory, 64-bit Windows 10 system. All experiments were implemented with the Keras framework TensorFlow backend using Python 3.7.

Since there were only 32 raw data samples in the private setting and 53 raw data samples in MODMA, the classification was not ideal in this case. Therefore, the intercepted data were divided by 1 s. To investigate the overall classification performance, the average and the standard deviation of the evaluation metrics were considered. The evaluation metrics used in this study were accuracy (*AC*), sensitivity (*SE*), specificity (*SP*) and the *F*1-score (*F*1), which are defined as follows:

(5) $$AC=\frac{TP+TN}{TP+FN+TN+FP^{\prime }}$$

(6) $$SE=\frac{TP}{TP+FN^{\prime }}$$

(7) $$SP=\frac{TN}{FP+TN^{\prime }}$$

(8) $$F1=\frac{2TP}{2TP+FP+FN^{\prime }}$$

where *TP* is the number of depression samples that are correctly classified, *FN* is the number of depression samples that are incorrectly classified as healthy samples, *FP* is the number of healthy samples that are incorrectly classified as depression cases, and *TN* is the number of healthy cases that are correctly classified.

Meanwhile, the binary cross-entropy loss function was used to measure the performance of the model.

## 3. Results

In this section, the performance of the proposed machine learning method to predict depression based on EEG signals is evaluated in several aspects. In the first part, the effect of the data augmentation procedure on the proposed framework is analyzed. Next, the obtained results of the proposed method are compared with the previous approaches. Finally, the proposed method was evaluated using another independent dataset, and its results are reported in the last subsection.

### 3.1. The Effect of Data Augmentation

In order to analyze the effect of data augmentation on the performance of the proposed method, EEG signals with different segment lengths were applied to the proposed classification framework. The proposed framework was tested with 1 s, 2 s and 3 s EEG segments. The obtained results of these simulations are reported in Table 2.

As shown in Table 2, the proposed method that included all of the data-augmentation strategies achieved a better classification performance and a considerable difference was not observed. Too many samples from one sample may not provide a good estimate of the generalization of the results. So, according to the result, it can be interpreted that the data augmentation procedure with 1s slicing led to the best classification performance.

### 3.2. The Influence of The number of CNN Layers

In order to quantify the benefits of a lower number of CNN layers, different layers were applied to the classification framework, including 1 layer, 2 layers and 3 layers. The obtained results of these simulations are reported in Table 3.

As shown in Table 3, due to the limited sample size, the increase in the number of CNN layers not only increased the time required but also significantly increased the number of parameters. When the number of layers increased to three, it can be seen that the accuracy decreased, which indicates over-fitting due to the depth of layers. So, according to the results, it can be interpreted that our model using a one-layer CNN network, which has the advantages of less time and lowers model complexity obtains better accuracy results. We can also infer that the proposed strategy is suitable for smaller data sets.

### 3.3. Comparison with Other Methods

In order to compare the performance of the proposed method with other approaches, we implemented the methods described in (S. Sun et al., (2020) [51]; Wang Y et al., (2021) [52]). For a fair comparison, all of the methods were validated using LOOCV. Table 4 provides the obtained numerical results of the proposed method as well as the previous ones for the automatic prediction of depression based on EEG signals of the MODMA dataset. Sun et al. [51] extracted different types of EEG features, including linear, nonlinear and functional connectivity features (Phase lag index, PLI), comprehensively analyzing the EEG signals of major depressive patients. Wang et al. [52] used an alternative time-frequency-analysis technique based on intrinsic time-scale decomposition (ITD) with TCN and L-TCN and obtained a better result. Compared with the above features, the brain-map-extracted frequency domain and spatial characteristics in the preprocessing of EEG signals was effective and achieved a better prediction performance for depression. Its accuracy was higher than in other studies, which demonstrates the effectiveness of the proposed strategy, as shown in Table 4.

Table 5 provides a comparison of the classification results between the different models with the MODMA dataset and the private dataset. According to the summarized results in Table 5, deep learning models are relatively better than SVM, and the time series model is more suitable for EEG signals. Moreover, we can see that the strategy for this work significantly improved compared to others both in accuracy, sensitivity, specificity and the *F*1 score.

## 4. Discussion

In this study, we proposed a novel EEG feature called the brain map, which contains temporal, frequency and spatial information, and compared the brain map with other features that have been applied in previous depression studies, including linear, nonlinear, PLI and ITD features. Moreover, previous studies about the discrimination between depression patients and normal subjects seldom considered spatial features based on a machine learning approach. In our study, we used the brain map feature with a frequency domain and spatial characteristics and found that it is able to achieve higher accuracy than other features. At present, the number of depression-related studies amounts to relatively few, and most of the research adopts the method of data segmentation to expand the data set. Table 2 provides a comparison of the classification results of the proposed method using the data-augmentation procedure. The proposed method with all of the data-augmentation strategies achieved a better classification performance and a considerable difference was not detected. However, an increase in sample size will increase the time required for data processing due to the projection of the operation from 3D to 2D during data preparation. So, we calculated features for a 1s segment to reduce the computation time. Table 3 shows that a one-layer CNN may achieve an accuracy rate of 87.98 percent. The number of CNN layers increases not only the time required but also the number of parameters by a significant amount. When the number of layers was increased to three, the accuracy declined, indicating over-fitting as a result of the depth of layers. Then, the addition of GRU introduced a special long-term memory in the CNN architecture to use sequence information. Table 5 shows that a CNN followed by a GRU network can increase network performance and improve the accuracy of the final prediction.

The proposed strategy has several advantages. First, it can achieve a higher classification accuracy than the existing methods for classification between depression patients and normal subjects which used the same MODMA dataset. Second, a low complexity network architecture (one layer CNN and one layer GRU) was applied, and according to the result, it can be interpreted that effectively extracted features during pre-processing could obtain good classification results using a simple neural network, especially for limitation samples.

The depression-prediction strategy was tested and validated with a private EEG dataset and a public EEG dataset. It was able to classify healthy and depressed patients with a very high accuracy of 89.63% for the MODMA dataset and 88.56% for the private dataset and effectively distinguish between depression and healthy individuals.

The automatic prediction method of depression proposed in this study not only solves the problem of the difficulty and low efficiency of manual diagnosis in the early clinical stage but also improves the prediction accuracy. As a simple and efficient method by which to measure the brain electrophysiological signal, the proposed strategy-based EEG feature seems to have the potential to assist psychiatrists in the diagnosis of depression.

## 5. Conclusions

This paper presents a signal preprocessing method by which to transform EEG signals into a brain map that contains temporal, frequency, and spatial information that attempts to make full use of the characteristics of EEG data. Then, a low complexity network architecture (one layer CNN and one layer GRU) was proposed to classify depression and healthy individuals. The lower CNN layer for limitation samples can help to overcome overfitting and improve the model’s generalization ability. The proposed strategy works well compared to other baseline models using the MODMA dataset.

Nevertheless, several follow-up studies will be required. First, only 53 subjects participated in the current study. To further validate the proposed method, more participants are needed in the future. Second, although the current results suggest that the frequency and spatial features are useful in the classification between depression patients and healthy controls, a combination of facial expressions, speech, and other features might achieve an even higher classification accuracy. Finally, the EEGs were recorded in the resting state in the current study. However, EEGs recorded in a different state may result in better performance for classifying depression patients and healthy controls. Moreover, the analysis in this study was mainly based on EEG data, and therefore its clinical interpretability is not very strong. In the future, we plan to cooperate with the hospital to enhance clinical interpretability with the help of expert knowledge.

## Figures and Tables

**Table 1:** Properties of datasets with data.

Properties	MODMA Dataset	Private Dataset
No. of participants	53	32
No. of depression cases	24	16
Depression diagnostics	Diagnosis	Diagnosis, BDI
Male/female ratio	33/20	16/16
No. of channels	128	16
Sampling rate, Hz	250	100

**Table 2:** Comparison of the classification results of the proposed method using the data augmentation procedure.

Method	Data Set	AC (Mean ± Std)	SE (Mean ± Std)	SP (Mean ± Std)	*F*1 (Mean ± Std)
1s slicing	MODMA	89.63 ± 1.3	90.24 ±1.9	89.63 ± 1.3	90.19 ± 1.3
	private dataset	88.56 ± 1.3	88.56 ± 1.5	88.54 ± 1.8	88.68 ± 1.5
2s slicing	MODMA	90.62 ± 2.1	87.81 ± 3.2	87.48 ± 2.1	88.79 ± 2.1
	private dataset	89.84 ± 2.1	87.82 ± 3.4	87.36 ± 1.7	88.79 ± 2.1
3s slicing	MODMA	87.01 ± 1.5	87.01 ± 1.5	87.01 ± 1.5	88.01 ± 1.5
	private dataset	87.72 ± 1.6	87.32 ± 1.6	86.72 ± 1.6	88.72 ± 1.6

**Table 3:** Comparison of the classification results between difference CNN layers.

Layers	Time (s)	Parameters	AC	SE	SP	*F*1
1	172	896	87.98	88.38	88.98	87.79
2	224	10,272	86.68	85.46	85.48	85.63
3	340	28,768	75.68	78.18	78.16	78.58

**Table 4:** Comparison of the classification results between the proposed method and previous works.

Methods	Features	Accuracy (%)
LR + ReliefF [51]	linear	66.40
LR + ReliefF [51]	nonlinear	67.17
LR + ReliefF [51]	PLI	82.31
LR + ReliefF [51]	Linear + PLI	80.99
LR + ReliefF [51]	Nonlinear + PLI	81.79
TCN [52]	ITD + statistical features	85.23
L-TCN [52]	ITD + statistical features	86.87
**BrainMap + CNN + GRU**	**BrainMap features**	**89.63**

**Table 5:** Comparison of the classification results between difference models.

Method	Dataset	AC	SE	SP	*F*1
SVM	MODMA	78.12	78.12	78.12	77.31
	Private dataset	75.18	74.92	75.12	74.31
GRU	MODMA	83.12	86.67	76.57	87.55
	Private dataset	81.36	82.49	78.91	82.55
CNN	MODMA	84.32	85.76	79.86	87.96
	Private dataset	82.34	84.35	79.91	83.31
TCN	MODMA	85.23	89.67	76.57	87.55
	Private dataset	82.38	82.47	82.47	82.55
L-TCN	MODMA	86.87	90.15	83.83	90.51
	Private dataset	85.64	85.87	81.23	86.55
BrainMap + CNN	MODMA	87.34	89.48	88.56	87.37
	Private dataset	83.65	82.59	82.31	82.55
**BrainMap + CNN + GRU**	**MODMA**	**89.63**	**90.24**	**89.63**	**90.19**
	**Private dataset**	**88.56**	**88.56**	**88.54**	**88.68**

## Data Availability

Publicly available datasets were analyzed in this study. This data can be found here: http://modma.lzu.edu.cn (accessed on 10 May 2021). The data presented in this study are available on request from the corresponding author.

## References

[1] World Health Organization (2017). Depression and Other Common Mental Disorders: Global Health Estimates.

[2] Friedrich M.J. (2017). Depression is the leading cause of disability around the world. JAMA.

[3] World Health Organization (2008). The Global Burden of Disease: 2004 Update.

[4] World Health Organization (2012). Depression: A Global Crisis.

[5] Preedy V.R., Watson R.R. (2010). International Classification of Disease.

[6] Arbanas G. (2015). Diagnostic and Statistical Manual of Mental Disorders (DSM-5).

[7] Jur Jurysta F., Kempenaers C., Lancini J., Lanquart J.P., Van De Borne P., Linkowski P. (2010). Altered interaction between cardiac vagal influence and delta sleep eeg suggests an altered neuroplasticity in patients suffering from major depressive disorder. Acta Psychiatr. Scand..

[8] Saeidi M., Karwowski W., Farahani F.V., Fiok K., Taiar R., Hancock P.A., Al-Juaid A. (2021). Neural Decoding of EEG Signals with Machine Learning: A Systematic Review. Brain Sci..

[9] Sharma M., Achuth P.V., Deb D., Puthankattil S.D., Acharya U.R. (2018). An Automated Diagnosis of Depression Using Three-Channel Bandwidth-Duration Localized Wavelet Filter Bank with EEG Signals. Cogn. Syst. Res..

[10] Liao S.C., Wu C.T., Huang H.C., Cheng W.-T., Liu Y.-H. (2017). Major Depression Detection from EEG Signals Using Kernel Eigen-Filter-Bank Common Spatial Patterns. Sensors.

[11] Bhat S., Acharya U.R., Hagiwara Y., Dadmehr N., Adeli H. (2018). Parkinson’s disease: Cause factors, measurable indicators, and early diagnosis. Comput. Biol. Med..

[12] Acharya U.R., Vinitha Sree S., Swapna G., Martis R.J., Suri J.S. (2013). Automated EEG analysis of epilepsy: A review. Knowl.-Based Syst..

[13] Kayser J., Tenke C.E. (2010). In search of the Rosetta Stone for scalp EEG: Converging on reference-free techniques. Clin. Neurophysiol..

[14] Acharya U.R., Hagiwara Y., Deshpande S.N., Suren S., Koh J.E.W., Oh S.L., Arunkumar N., Ciaccio E.J., Lim C.M. (2019). Characterization of focal EEG signals: A review. Future Gener. Comput. Syst..

[15] Gu X., Yang B., Gao S., Yan L.F., Xu D., Wang W. (2021). Application of bi-modal signal in the classification and recognition of drug addiction degree based on machine learning. Math. Biosci. Eng..

[16] Gao Y., Cao Z., Liu J., Zhang J. (2021). A novel dynamic brain network in arousal for brain states and emotion analysis. Math. Biosci. Eng..

[17] Wei Y. (2005). Comparative analysis of electroencephalogram in patients with neurological disorders and depression. J. Shanxi Med. Univ..

[18] Siuly S., Li Y., Zhang Y. (2016). EEG signal analysis and classification. IEEE Trans. Neural. Syst. Rehabil. Eng..

[19] Campisi P., La Rocca D. (2014). Brain waves for automatic biometric-based user recognition. IEEE Trans. Inform. Forensics Secur..

[20] Kumar J.S., Bhuvaneswari P. (2012). Analysis of electroencephalography (EEG) signals and its categorization–A study. Procedia Eng..

[21] Novik O., Smirnov F., Volgin M. (2019). Structures of the brain. Electromagnetic Geophysical Fields.

[22] Khosla A., Khandnor P., Chand T. (2020). A comparative analysis of signal processing and classification methods for different applications based on EEG signals. Biocybern. Biomed. Eng..

[23] Bachmann M., Päeske L., Kalev K., Aarma K., Lehtmets A., Ööpik P., Lass J., Hinrikus H. (2018). Methods for classifying depression in single channel EEG using linear and nonlinear signal analysis. Comput. Methods Programs Biomed..

[24] Koller-Schlaud K., Ströhle A., Bärwolf E., Behr J., Rentzsch J. (2020). EEG frontal asymmetry and theta power in unipolar and bipolar depression. J. Affect. Disord..

[25] Kang M., Kwon H., Park J.H., Kang S., Lee Y. (2020). Deep-asymmetry: Asymmetry matrix image for deep learning method in pre-screening depression. Sensors.

[26] Liu W., Zhang C., Wang X., Xu J., Chang Y., Ristaniemi T., Cong F. (2020). Functional connectivity of major depression disorder using ongoing EEG during music perception. Clin. Neurophysiol..

[27] Grin-Yatsenko V.A., Baas I., Ponomarev V.A., Kropotov J.D. (2010). Independent component approach to the analysis of EEG recordings at early stages of depressive disorders. Clin. Neurophysiol..

[28] Heinsfeld A.S., Franco A.R., Craddock R.C., Buchweitz A., Meneguzzi F. (2018). Identification of autism spectrum disorder using deep learning and the ABIDE dataset. Neuroimage Clin..

[29] Grotegerd D., Suslow T., Bauer J., Ohrmann P., Arolt V., Stuhrmann A., Heindel W., Kugel H., Dannlowski U. (2013). Discriminating unipolar and bipolar depression by means of fMRI and pattern classification: A pilot study. Eur. Arch. Psychiatry Clin. Neurosci..

[30] Kermany D.S., Goldbaum M., Cai W., Valentim C.C.S., Liang H., Baxter S.L., McKeown A., Yang G., Wu X., Yan F. (2018). Identifying medical diagnoses and treatable diseases by image-based deep learning. Cell.

[31] Hannesdóttir D.K., Doxie J., Bell M.A., Ollendick T.H., Wolfe C.D. (2010). A longitudinal study of emotion regulation and anxiety in middle childhood: Associations with frontal EEG asymmetry in early childhood. Dev. Psychobiol..

[32] Avram J., Baltes F.R., Miclea M., Miu A.C. (2010). Frontal EEG activation asymmetry reflects cognitive biases in anxiety: Evidence from an emotional face Stroop task. Appl. Psychophysiol. Biofeedback.

[33] Thibodeau R., Jorgensen R.S., Kim S. (2006). Depression, anxiety, and resting frontal EEG asymmetry: A meta-analytic review. Abnorm. Psychol..

[34] Hosseinifard B., Moradi M.H., Rostami R. (2013). Classifying depression patients and normal subjects using machine learning techniques and nonlinear features from EEG signal. Comput. Methods Programs Biomed..

[35] Field T., Diego M. (2008). Maternal depression effects on infant frontal EEG asymmetry. Int. J. Neurosci..

[36] Iosifescu D.V., Greenwald S., Devlin P., Mischoulon D., Denninger J.W., Alpert J., Fava M. (2009). Frontal EEG predictors of treatment outcome in major depressive disorder. Eur. Neuropsychopharmacol..

[37] Bisch J., Kreifelts B., Bretscher J., Wildgruber D., Fallgatter A., Ethofer T. (2016). Emotion perception in adult attention-deficit hyperactivity disorder. Neural Transm..

[38] Yahya N., Musa H., Zhong Y.O., Ong Z.Y., Elamvazuthi I. (2019). Classification of Motor Functions from Electroencephalogram (EEG) Signals Based on an Integrated Method Comprised of Common Spatial Pattern and Wavelet Transform Framework. Sensors.

[39] Ahmadlou M., Adeli H., Adeli A. (2012). Fractality analysis of frontal brain in major depressive disorder. Int. J. Psychophysiol..

[40] Faust O., Ang P.C.A., Puthankattil S.D., Joseph P.K. (2014). Depression diagnosis support system based on EEG signal entropies. Mech. Med. Biol..

[41] Bairy G.M., Niranjan U., Puthankattil S.D. (2016). Automated classification of depression EEG signals using wavelet entropies and energies. Mech. Med. Biol..

[42] Acharya U.R., Oh S.L., Hagiwara Y., Tan J.H., Adeli H., Subha D.P. (2018). Automated EEG-based screening of depression using deep convolutional neural network. Comput. Methods Programs Biomed..

[43] Ay B., Yildirim O., Talo M., Baloglu U.B., Aydin G., Puthankattil S.D., Acharya U.R. (2019). Automated depression detection using deep representation and sequence learning with EEG signals. Med. Syst..

[44] Sharma G., Parashar A., Joshi A.M. (2021). DepHNN: A novel hybrid neural network for electroencephalogram (EEG)-based screening of depression. Biomed. Signal Processing Control.

[45] Cai H., Gao Y., Sun S., Li N., Tian F., Xiao H., Li J., Yang Z., Li X., Zhao Q. (2020). MODMA dataset: A multi-modal open dataset for mental disorder analysis. arXiv.

[46] Almars A.M. (2022). Attention-Based Bi-LSTM Model for Arabic Depression Classification. CMC-Comput. Mater. Contin..

[47] Li X., Zhang X., Zhu J., Mao W., Sun S., Wang Z., Xia C., Hu B. (2019). Depression recognition using machine learning methods with different feature generation strategies. Artif. Intell. Med..

[48] Ahmad A. (2014). 3D to 2D bijection for spherical objects under equidistant fisheye projection. Comput. Vis. Image Underst..

[49] Wang Z., Du X., Wu Q., Dong Y. Research on the multi-classifier features of the motor imagery EEG signals in the brain computer interface. Proceedings of the Tenth International Conference on Digital Image Processing (ICDIP 2018), International Society for Optics and Photonics.

[50] Cho K., Van Merrienboer B., Gulcehre C., Bahdanau D., Bougares F., Schwenk H., Bengio Y. Learning Phrase Representations using RNN Encoder-Decoder for Statistical Machine Translation. Proceedings of the Empiricial Methods in Natural Language Processing (EMNLP 2014).

[51] Shuting S., Jianxiu L., Huayu C., Tao G., Xiaowei L., Bin H. (2020). A study of resting-state EEG biomarkers for depression recognition. arXiv.

[52] Wang Y., Liu F., Yang L. EEG-Based Depression Recognition Using Intrinsic Time-scale Decomposition and Temporal Convolution Network. Proceedings of the Fifth International Conference on Biological Information and Biomedical Engineering.

